# RNA sequencing data of human periodontal ligament cells treated with continuous and intermittent compressive force

**DOI:** 10.1016/j.dib.2019.104553

**Published:** 2019-09-23

**Authors:** Jeeranan Manokawinchoke, Prasit Pavasant, Chenphop Sawangmake, Nuttapol Limjeerajarus, Chalida N. Limjeerajarus, Hiroshi Egusa, Thanaphum Osathanon

**Affiliations:** a Center of Excellence for Regenerative Dentistry and Department of Anatomy, Faculty of Dentistry, Chulalongkorn University, Bangkok, 10330, Thailand; b Department of Pharmacology, Faculty of Veterinary Science, Chulalongkorn University, Bangkok, 10330, Thailand; c Research Center for Advanced Energy Technology, Faculty of Engineering, Thai-Nichi Institute of Technology, Bangkok 10250, Thailand; d Department of Physiology, Faculty of Dentistry, Chulalongkorn University, Bangkok, 10330, Thailand; e Division of Molecular and Regenerative Prosthodontics, Tohoku University Graduate School of Dentistry, Sendai 980-8575, Japan; f Genomics and Precision Dentistry Research Unit, Faculty of Dentistry, Chulalongkorn University, Bangkok, 10330, Thailand

**Keywords:** Periodontal ligament, Mechanical force, RNA sequencing

## Abstract

Mechanical force regulates numerous biological functions. Application of different force types leads to different cell responses. This data article describes RNA sequencing data identifying gene expression of human periodontal ligament cells (hPDLs) treated with the continuous or intermittent compressive force. These data could be further utilized to investigate the controlling pathways that regulate hPDLs’ behaviors by the different force types. Raw RNA sequencing data were deposited in the NCBI Sequence Read Archive (SRP136155) and NCBI Gene Expression Omnibus (GSE112122).

**Table:** Specifications Table

Subject area	Biology
More specific subject area	Oral biology
Type of data	FASTQ file, Tables, Figures
How data was acquired	RNA sequencing
Data format	Raw data
Experimental factors	The computerized controlled continuous and intermittent compressive forces.
Experimental features	hPDLs were treated with the computerized controlled continuous or intermittent compressive force for 24 hours in serum-free culture condition. Cells without mechanical treatment were used as the control. After the total RNA was isolated, the quality of mRNA was determined and mRNA was further processed for library preparation. Subsequently, gene expression profiles were analyzed using a high throughput RNA sequencing with NextSeq 500 (Illumina).
Data source location	Bangkok, Thailand
Data accessibility	Raw data generated from sequencing were deposited at NCBI Sequence Read Archive (SRP136155) https://www.ncbi.nlm.nih.gov/sra?term=SRP136155 and the processed read counts of gene expression were deposited at NCBI Gene Expression Omnibus (GSE112122). https://www.ncbi.nlm.nih.gov/geo/query/acc.cgi?acc=GSE112122
Related research article	J. Manokawinchoke, P. Pavasant, C. Sawangmake, N. Limjeerajarus, C. Limjeerajarus, H. Egusa, T. Osathanon, Intermittent compressive force promotes osteogenic differentiation in human periodontal ligament cells by regulating the transforming growth factor beta pathway, Cell Death and Disease (2019).

**Table:** 

Value of the Data • Gene expression data could be further investigated to reveal the regulatory pathways and mechanisms related to the influence of mechanical force on hPDLs' behaviors. • Researchers in orthodontics and periodontics related areas may utilize these data to identify regulatory mechanism(s) by which force controls hPDLs' functions and responses. • Specific pathways can be identified to determine different regulatory mechanism of different force types on hPDLs' biological responses. • Meta-analysis can be performed with other related databases to increase statistical power of the investigation for identification of genes regulated by mechanical force.

## 1 Data

**Fig. 1:**
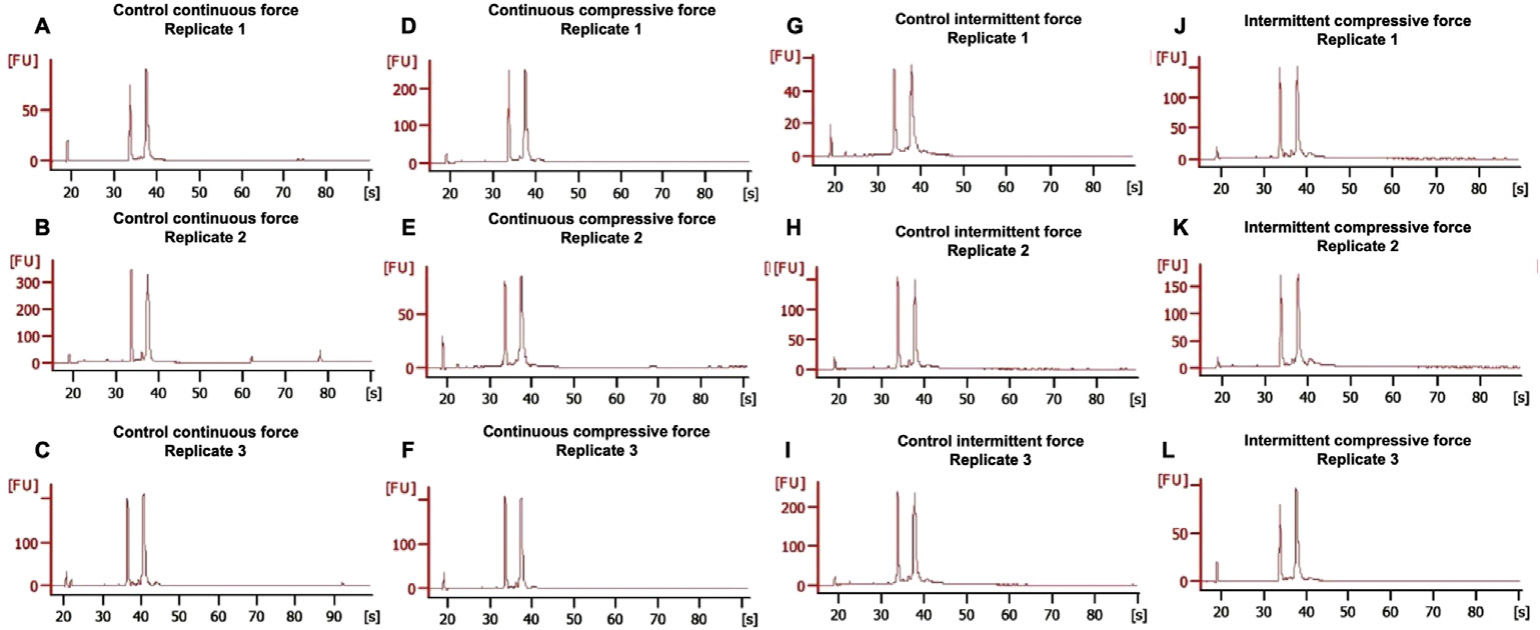
RNA quality was checked by Bioanalyzer. (A–C) The unloaded control for the continuous compressive force treatment; (D–F) the continuous compressive force treatment; (G–I) the unloaded control for the intermittent compressive force treatment; (J–L) the intermittent compressive force treatment.

**Fig. 2:**
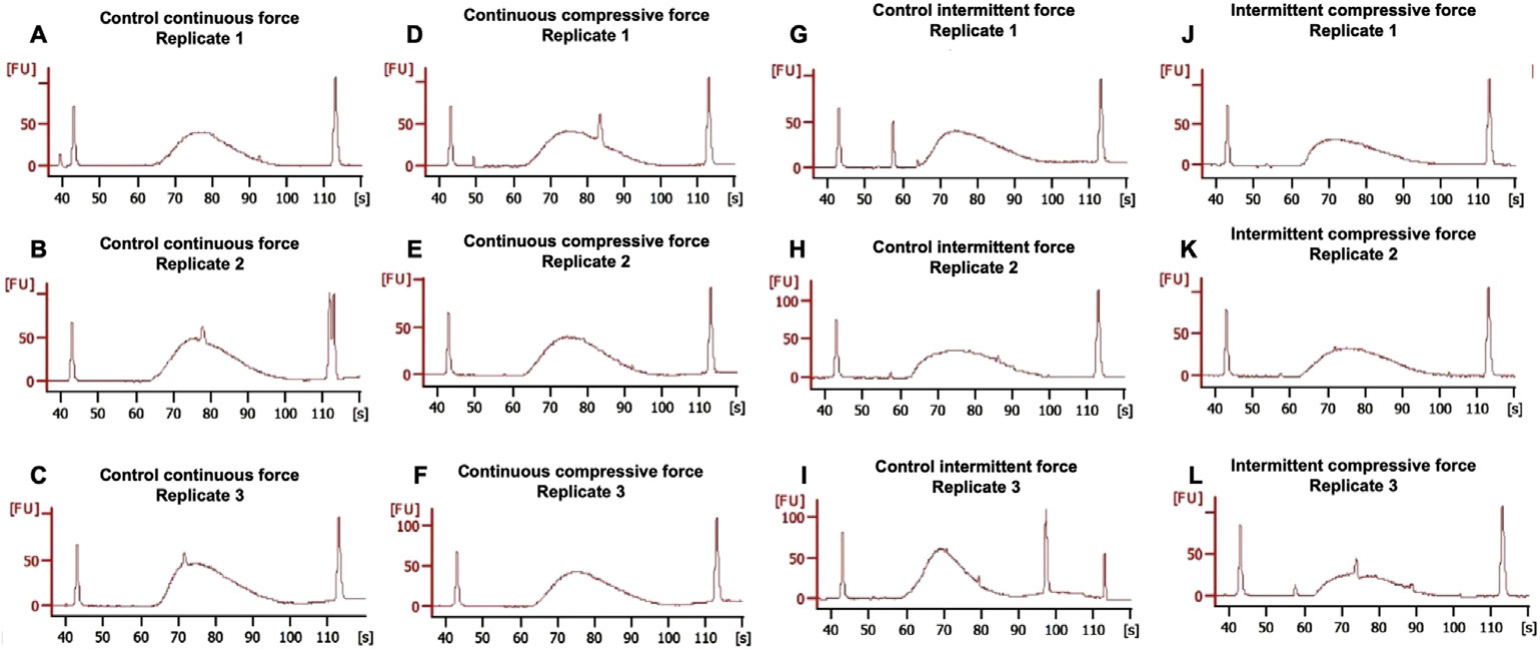
Quality and size of library was evaluated using Bioanalyzer. (A–C) The unloaded control for the continuous compressive force treatment; (D–F) the continuous compressive force treatment; (G–I) the unloaded control for the intermittent compressive force treatment; (J–L) the intermittent compressive force treatment.

Mechanical force regulates numerous cell functions [1], [2]. Application of different force types leads to the different cell responses [2]. Periodontal ligament is always subjected to mechanical force during normal function for example chewing. This data article described the gene expression profiles of human periodontal ligament cells (hPDLs) after treating with the continuous or intermittent compressive force using RNA sequencing technique. The isolated RNA demonstrated the high intact and quality RNA input as shown by RNA integrity number higher than 9.0 (Fig. 1). After library preparation, average library concentration and size of samples were in the range of 89–231 nM and 248–293 base pair, respectively (Table 2). Library quality assurance was conducted using bioanalyzer (Fig. 2). RNA sequencing was performed using NextSeq500 (Illumina). Ninety four percent of reads exhibited Q score higher than 30 (Table 3). Average number of reads was ranged from 30.6 to 37.1 million reads (75 bp; single-end). Reads exhibited total alignment percentage higher than 96% and base calling error rate was as low as 0.21% (Table 4).

**Table 1:** Information of samples for differential gene expression of RNA sequencing analysis of the mechanical compressive forces treated human periodontal ligament cells.

Replicate	Source	Protocol 1	Protocol 2	Protocol 3	Sequencer	Read length (bp)	GEO accession number
1	Human periodontal ligament cells	Control unloaded continuous compressive force	Total RNA extraction	RNA-Seq	Illumina NextSeq 500	75 reads forward-end	GSM3058133
1	Human periodontal ligament cells	Continuous compressive force	Total RNA extraction	RNA-Seq	Illumina NextSeq 500	75 reads forward-end	GSM3058136
1	Human periodontal ligament cells	Control unloaded intermittent compressive force	Total RNA extraction	RNA-Seq	Illumina NextSeq 500	75 reads forward-end	GSM3058139
1	Human periodontal ligament cells	Intermittent compressive force	Total RNA extraction	RNA-Seq	Illumina NextSeq 500	75 reads forward-end	GSM3058142
2	Human periodontal ligament cells	Control unloaded continuous compressive force	Total RNA extraction	RNA-Seq	Illumina NextSeq 500	75 reads forward-end	GSM3058134
2	Human periodontal ligament cells	Continuous compressive force	Total RNA extraction	RNA-Seq	Illumina NextSeq 500	75 reads forward-end	GSM3058137
2	Human periodontal ligament cells	Control unloaded intermittent compressive force	Total RNA extraction	RNA-Seq	Illumina NextSeq 500	75 reads forward-end	GSM3058140
2	Human periodontal ligament cells	Intermittent compressive force	Total RNA extraction	RNA-Seq	Illumina NextSeq 500	75 reads forward-end	GSM3058143
3	Human periodontal ligament cells	Control unloaded continuous compressive force	Total RNA extraction	RNA-Seq	Illumina NextSeq 500	75 reads forward-end	GSM3058135
3	Human periodontal ligament cells	Continuous compressive force	Total RNA extraction	RNA-Seq	Illumina NextSeq 500	75 reads forward-end	GSM3058138
3	Human periodontal ligament cells	Control unloaded intermittent compressive force	Total RNA extraction	RNA-Seq	Illumina NextSeq 500	75 reads forward-end	GSM3058141
3	Human periodontal ligament cells	Intermittent compressive force	Total RNA extraction	RNA-Seq	Illumina NextSeq 500	75 reads forward-end	GSM3058144

**Table 2:** Average library size and concentration.

Sample ID	Library concentration (nM)	Average library size (bp)
Control unloaded continuous force Replicate 1	109	293
Control unloaded continuous force Replicate 2	201	292
Control unloaded continuous force Replicate 3	228	290
Continuous compressive force Replicate 1	140	286
Continuous compressive force Replicate 2	160	290
Continuous compressive force Replicate 3	222	289
Control unloaded intermittent force Replicate 1	209	279
Control unloaded intermittent force Replicate 2	152	269
Control unloaded intermittent force Replicate 3	231	248
Intermittent compressive force Replicate 1	151	257
Intermittent compressive force Replicate 2	112	284
Intermittent compressive force Replicate 3	89	280

**Table 3:** NextSeq run summary.

Read	Error rate (%)	Cluster Passing Filter (%)	Read Passing Filter (millions)	Q score >30 (%)
Read 1 (Forward-end)	0.21	95.70	400	94.92

**Table 4:** RNA-Seq alignment summary.

Sample ID	Read length	Number of reads (million)	Total aligned (%)
Control unloaded continuous force Replicate 1	75	30.8	97.73
Control unloaded continuous force Replicate 2	75	32.9	97.89
Control unloaded continuous force Replicate 3	75	37.1	97.81
Continuous compressive force Replicate 1	75	31.3	97.91
Continuous compressive force Replicate 2	75	35.0	96.52
Continuous compressive force Replicate 3	75	35.4	97.41
Control unloaded intermittent force Replicate 1	75	36.5	97.45
Control unloaded intermittent force Replicate 2	75	31.5	96.88
Control unloaded intermittent force Replicate 3	75	31.7	97.47
Intermittent compressive force Replicate 1	75	30.6	97.21
Intermittent compressive force Replicate 2	75	30.7	96.65
Intermittent compressive force Replicate 3	75	32.2	97.77

## 2 Experimental design, materials and methods

Methods described in the following section are expanded version from our related work [3].

### 2.1 Cell isolation and culture

Experiment was approved by the Human Ethics Committee, Faculty of Dentistry, Chulalongkorn University (Study code HREC-DCU 2018-001). Periodontal tissues were gently scraped from the middle area of the tooth's root. Cell isolation was performed by the explant protocol. Growth medium was Dulbecco's Modified Eagle's Medium (Gibco, Carlsbad, CA, USA) with the addition of with 10% fetal bovine serum (Gibco), 2mM l-glutamine (Invitrogen, Carlsbad, CA, USA), 100 Units/ml penicillin (Invitrogen), 100 μg/ml streptomycin (Invitrogen), and 250 ng/ml amphotericin B (Invitrogen). The isolated cells were cultured at 37 °C in a humidified 5% CO_2_ atmosphere.

### 2.2 Compressive force treatment

Cell were subjected to mechanical compressive force using a computer-controlled apparatus [1], [4]. Briefly, cells (37,500 cells/cm^2^) were plated in 6-well tissue culture plates and maintained in growth medium for 24 h. After the serum starvation for 8 h, cells were treated to continuous or intermittent compressive force, according to previous publications [1], [4]. In brief, cells were continuously loaded with 1.5 g/cm^2^ force for a continuous force treatment. For intermittent compressive force application, cells were loaded with 1.5 g/cm^2^ force at frequency of 0.23 Hz.

### 2.3 RNA preparation and sequencing

Cells were loaded with the continuous or intermittent compressive force in serum free culture condition for 24 h. The unloaded cells were employed as the control. Total cellular RNA was extracted using a RNeasy Plus Mini Kit with DNaseI treatment (Qiagen, USA). Each group consisted of the samples from three independent individuals (Table 1). RNA sequencing and bioinformatic analyses were performed and evaluated at the Omics Science and Bioinformatics Center, Faculty of Science, Chulalongkorn University. RNA quality and quantity were determined using a Nanodrop and a bioanalyzer (Aligent 2100; Agilent Technologies, Santa Clara, CA, USA). Nanodrop analysis revealed that the extracted RNA exhibited an OD260/280 ratio of 2.06–2.09 and the OD260/230 ratio ranged from 1.58 to 1.91. The RNA concentration ranged from 141.9 to 165.5 ng/μl. Further, mRNA library was prepared using the TrueSeq mRNA stranded library preparation kit (Illumina, San Diego, CA, USA). TrueSeq adapter-index was ligated to cDNA libraries and subsequently library enrichment was performed using polymerase chain reaction amplification for 8 cycles. Bioanalyzer was employed to determine RNA integrity number (RIN) (Fig. 1) and sequencing library quality (Fig. 2). Qubit 3.0 fluorometer (Thermo Fisher Scientific, Waltham, MA, USA) was used to evaluate library size and concentration (Table 2). NextSeq500 (Illumina) was employed for sequencing analysis.

### 2.4 Quality validation and read mapping

**Fig. 3:**
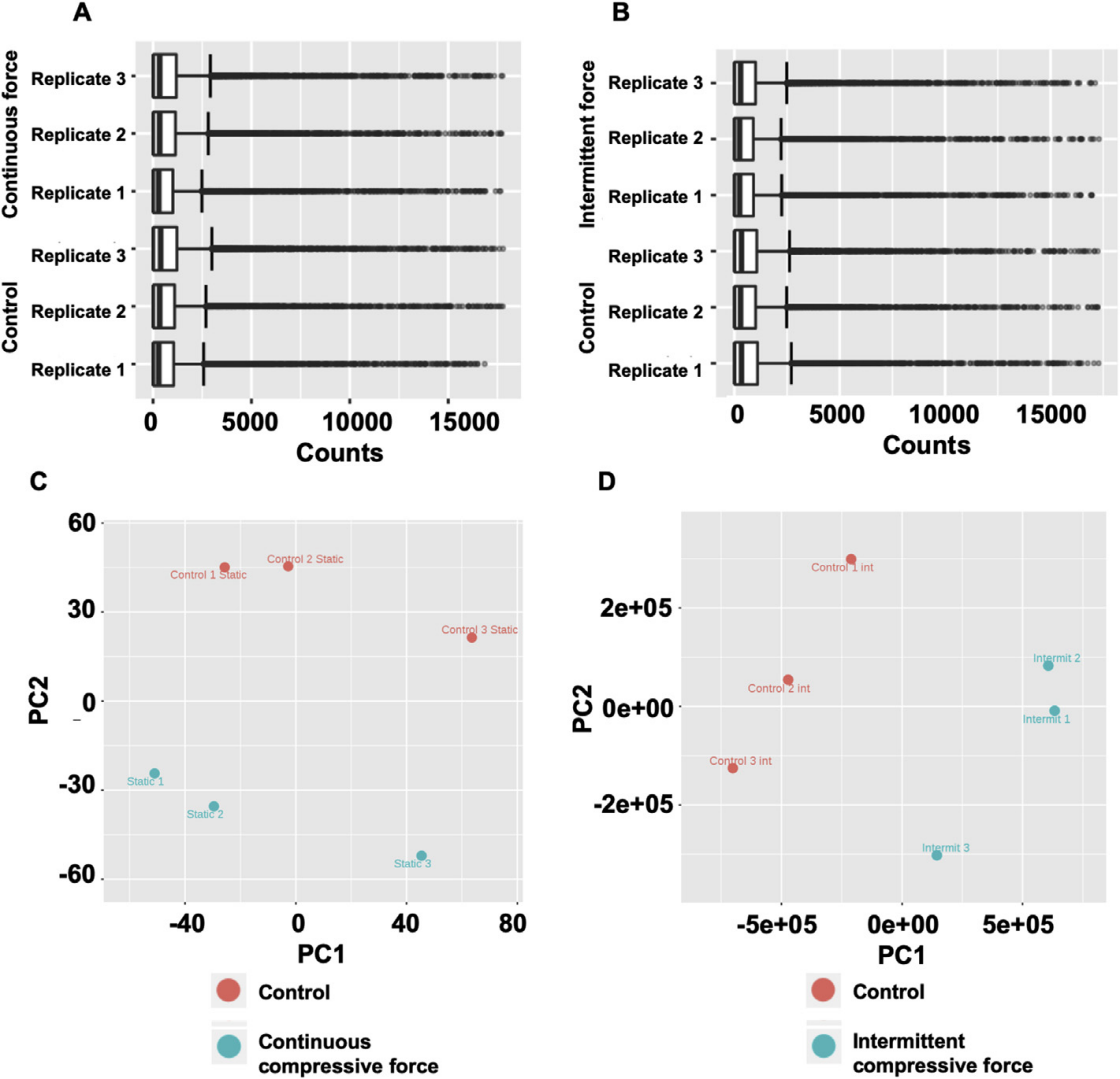
The distribution of raw read counts for the continuous (A) and the intermittent (B) compressive force experiment. Variance of samples was examined using principle component analysis diagram (PCA) for the continuous (C) and the intermittent (D) compressive force experiment.

**Fig. 4:**
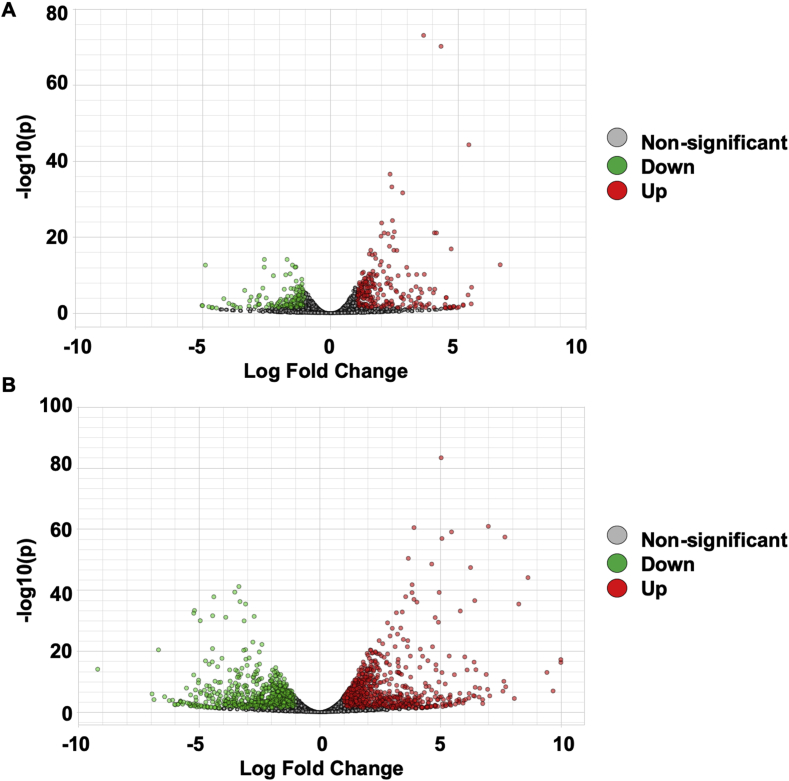
Volcano plots demonstrated the up- and down-regulated genes in the continuous (A) and intermittent (B) compressive force treated cells compared with the control.

RTA2 software was used to analyze base calling and Q scoring. A bcl2fastq software was employed for file conversion and demultiplexing. FastQC and Trimmomatic were utilized to check read quality [5], [6]. Trimmomatic was also employed for read trimming and filtering [5], [6]. *Homo sapiens UCSC hg38* was used as the reference for read mapping by HISAT2 [7]. Transcript quantification was performed using HTseq count [8]. The NextSeq run summary was shown in Table 3. Total alignment of each samples was demonstrated in Table 4. The distribution of raw read count was demonstrated (Fig. 3A and B). Variance was determined using principle component analysis (Fig. 3C and D). Further, the differential gene expression was determined using EdgeR [9], [10]. Genes that exhibited the Log2 fold change ≥1.0 or ≤1.0 were included. Significant difference was considered when FDR <0.05. Fig. 4 illustrated the volcano plots of up- and down-regulated genes in the continuous and intermittent compressive force treated cells compared with the control.
